# Assessing changing weather and the El Niño Southern Oscillation impacts on cattle rabies outbreaks and mortality in Costa Rica (1985–2016)

**DOI:** 10.1186/s12917-018-1588-8

**Published:** 2018-09-17

**Authors:** Sabine E. Hutter, Annemarie Käsbohrer, Silvia Lucia Fallas González, Bernal León, Katharina Brugger, Mario Baldi, L. Mario Romero, Yan Gao, Luis Fernando Chaves

**Affiliations:** 1 Institute of Veterinary Public Health, Department for Farm Animals and Veterinary Public Health, University of Veterinary Medicine, Veterinärplatz 1, 1210 Wien, Austria; 2 Laboratorio de Pruebas de Paternidad, Caja Costarricense del Seguro Social (CCSS), San José, Costa Rica; 3 Servicio Nacional de Salud Animal (SENASA), Heredia, Costa Rica; 4 Research Institute of Wildlife Ecology, University of Veterinary Medicine, Vienna, Austria; 5 Centro de Investigación en Enfermedades Tropicales (CIET), Universidad de Costa Rica, San Pedro de Montes de Oca, Costa Rica; 6 Centro de Investigaciones en Geografía Ambiental, Universidad Nacional Autónoma de México, 58190 Morelia, Michoacán Mexico; 7 Instituto Costarricense de Investigación y Enseñanza en Nutrición y Salud, Apartado Postal 4-2250, Tres Ríos, Cartago, Costa Rica; 8 Programa de Investigación en Enfermedades Tropicales (PIET), Escuela de Medicina Veterinaria, Universidad Nacional, Heredia, Costa Rica

**Keywords:** *Bos taurus*, Costa Rica, Rhabdovirus, Climate change, Wavelets

## Abstract

**Background:**

Rabies is a major zoonotic disease affecting humans, domestic and wildlife mammals. Cattle are the most important domestic animals impacted by rabies virus in the New World, leading to thousands of cattle deaths per year and eliciting large economic losses. In the New World, virus transmission in cattle is primarily associated with *Desmodus rotundus*, the common vampire bat. This study analyses the association of weather fluctuations and the El Niño Southern Oscillation (ENSO), with the occurrence and magnitude, in terms of associated mortality, of cattle rabies outbreaks. Data from the 100 cattle rabies outbreaks recorded between 1985 and 2016 in Costa Rica were analyzed. Periodograms for time series of rabies outbreaks and the El Niño 4 index were estimated. Seasonality was studied using a seasonal boxplot. The association between epidemiological and climatic time series was studied via cross wavelet coherence analysis. Retrospective space-time scan cluster analyses were also performed. Finally, seasonal autoregressive time series models were fitted to study linear associations between monthly number of outbreaks, monthly mortality rates and the El Niño 4 index, temperature, and rainfall.

**Results:**

Large rabies mortality occurred towards the Atlantic basin of the country. Outbreak occurrence and size were not directly associated with ENSO, but were sensitive to weather variables impacted by ENSO. Both, ENSO phases and rabies outbreaks, showed a similar 5 year period in their oscillations. Cattle rabies mortality and outbreak occurrence increased with temperature, whereas outbreak occurrence decreased with rainfall. These results suggest that special weather conditions might favor the occurrence of cattle rabies outbreaks.

**Conclusions:**

Further efforts are necessary to articulate the mechanisms underpinning the association between weather changes and cattle rabies outbreaks. One hypothesis is that exacerbation of cattle rabies outbreaks might be mediated by impacts of weather conditions on common vampire bat movement and access to food resources on its natural habitats. Further eco-epidemiological field studies could help to understand rabies virus transmission ecology, and to propose sound interventions to control this major veterinary public health problem.

**Electronic supplementary material:**

The online version of this article (10.1186/s12917-018-1588-8) contains supplementary material, which is available to authorized users.

## Background

Rabies is a major zoonotic disease worldwide, with a high human and domestic animal death toll. Rabies virus is a negative-sense single stranded ribonucleic acid (RNA) virus belonging to the *Lyssavirus* genus in the Rhabdoviridae family [1]. Recently, it was estimated that globally canine rabies causes approximately 59,000 human deaths [2]. Furthermore, over 99% of all virus transmission to humans comes from dogs, the rest mainly coming from bats [3].

The problem of humans being at risk of rabies virus transmission has existed in the New World for centuries [4, 5]. Nowadays, due to an elimination programme for dog transmitted human rabies initiated in 1983 [6, 7], the main mode of zoonotic rabies transmission in the New World is via bites from infected common vampire bats to other mammal species, including humans and livestock [3]. This was also the case in Costa Rica, where dog rabies has not been a problem for decades [8].

The common vampire bat, *Desmodus (D.) rotundus*, is the major reservoir and vector of rabies virus in the New World [9–14]. *D. rotundus* bloodfeeds on mammals and transmits rabies virus, with livestock animals being greatly affected by this lethal disease [15]. In fact, common vampire bat bites and rabies transmission are limiting factors for livestock, mainly cattle production, as shown in an economic evaluation for Mexico [6, 11]. In 1968 it was estimated that over 500,000 cattle died from bat-transmitted rabies in Latin America [16]. Following the establishment of bat control methods and cattle rabies vaccination campaigns, rabies virus associated cattle mortality has plummeted [6, 16]. By 1983, rabies was responsible for 9904 cattle deaths, for 1831 in 1993 and for 1580 in 2006 in Latin America [6, 16]. Nevertheless, in Latin America, the decline has not been always monotonic, with surges in rabies virus associated cattle mortality, like in 2000, when 6088 cattle heads died by rabies, and in 2002, when cattle deaths totaled 3327 animals [13, 16]. The economic loss associated with cattle deaths by rabies has also been significant all over Latin America, adding to an estimate of 100 million US dollars in 1966 [17] and between 44 and 50 million US dollars by 1986 [18].

The epidemiology of numerous cattle rabies outbreaks has been studied across Latin America [4, 7, 9], in places so far apart like northeastern Mexico [19] and southern Brazil [20]. In Costa Rica, between 1985 and 2014, over 70 cattle rabies outbreaks were reported, including 723 cattle deaths, placing a considerable burden on the local livestock industry [8]. All of these outbreaks have been linked by the unequivocal presence of common vampire bats, and this might be enhanced by a couple of factors. The first factor is that common vampire bat dispersal is directly linked with a preference for bovine meals, probably because fenced-in cattle are a predictable resource when compared with free-ranging natural hosts [21]. This situation generates perfect conditions for transmission in domestic animals not observed in wildlife species or in free range grazing livestock herds [22]. The second factor is that common vampire bats are organisms sensitive to weather changes, despite having an endothermic nature. For example, it is known that temperature influences the geographic distribution of the common vampire bat [23]. Common vampire bats tend to be limited to low elevations in the tropics, mainly because of food availability, but also because of limitations on the maximum meal weight they can carry in flight, and the low energetic cost for keeping common vampire bat body temperature constant [23]. Common vampire bats move between caves depending on the presence of roosts and climatic conditions [24]. For example, high humidity seems essential for common vampire bat roosting, in dry areas common vampire bats roost in water wells and leave the wells as they become dry, concentrating in wells with water during the dry season, and dispersing to other wells as they get filled with water during the rainy season [25]. Moreover, foraging time [26] and roost size [24] of common vampire bats seem to follow seasonal patterns. For example, common vampire bats spend more time attached to their prey during the dry season than they do during the wet season, likely ingesting more blood during the dry season [26]. Although controversial [27, 28], the birth of common vampire bats seems to be seasonal, despite the fixed 205 day gestation period recorded for vampires. However, when vampire births occur during the rainy season, rabies transmission could be enhanced via increased bat movement [25]. Nevertheless, little attention has been given to the potential impacts that anomalous weather conditions could have on the likelihood of cattle rabies outbreaks, despite abundant observations have shown that common vampire bat biology, and rabies transmission, could be sensitive to environmental changes.

ENSO can lead to extreme climatic conditions, such as severe droughts and floods, and more generally can alter weather patterns in a manner that creates ideal ecological conditions for disease transmission [29–31]. In Central America, several studies have found a differential impact of ENSO on the incidence of malaria [32, 33] and leishmaniasis [34], making this region ideal to test hypotheses about differential ENSO impacts on disease transmission. To the best of our knowledge, only one study has analysed rabies cases in relation to ENSO, where 416 human rabies cases mainly associated to dogs were recorded in Venezuela during 2002–2004 [35]. This study showed an increased human rabies incidence during the cold ENSO phase (La Niña in 2004) when compared to the hot ENSO phase (El Niño in 2002) [35], a suggestive observation despite data limitations for statistical inferences. Thus, provided that global climate change, here defined as the increase in frequency and intensity of extreme weather events driven by phenomena like ENSO, is known to affect livestock infectious diseases [36], such as Rift Valley Fever, and many other infectious diseases [37, 38] and the existence of detailed records about cattle rabies outbreaks in Costa Rica [8] creates an ideal scenario to test the hypothesis that ENSO phases might have differential impacts on the occurrence and size of cattle rabies outbreaks. Here, it is studied if the frequency of rabies outbreaks and cattle mortality per outbreak are associated with ENSO driven changes in weather patterns in Costa Rica between 1985 and 2016.

## Methods

### Study area

Costa Rica is located in Central America, bordering Nicaragua to the North, Panama to the Southeast, the Pacific Ocean to the West, and the Caribbean Sea (Atlantic Ocean) to the East. This small country of 51,100 km^2^ has a tropical climate. According to the last agricultural census, in 2014, there were around 37,000 cattle farms with 1,300,000 cattle heads in Costa Rica [39]. The number of cattle heads has decreased 37.5% since the last census in 1984. Several factors have driven this herd size decline, including a cattle crisis in the 1980s, mainly driven by a price reduction for meat [40]. In addition, the creation of national parks and biological reserves since the 1970s, in accordance with Costa Rica’s land use development strategy, promoted land use change from cattle ranching to biodiversity conservation areas [41, 42].

### Epidemiological data

Data on cattle rabies cases from 1985 to 2016 from Servicio Nacional de Salud Animal (SENASA), the Costa Rican Veterinary Authority were used. Their database includes the data of all registered outbreaks, the number and species of dead animals attributed to rabies, and the georeferenced location of the outbreak. Meanwhile, the geolocation of outbreaks was variable, as in some instances it was based on the centroid of the county where the outbreak happened, while in other instances it was based on the coordinates of the cattle ranch itself, measured with a Global Positioning System (GPS) [8].

Detailed information on rabies diagnostic testing was available from the laboratory database [8]. During the study period diagnostic tools became more robust by the inclusion of more sensitive and specific methods. Starting in 1983 with the detection of Negri bodies in brain samples processed with Sellers staining, the diagnostic method changed to mice inoculation and immune fluorescence testing in 1993. The latter method was enhanced by the addition of several deoxyribonucleic acid (DNA) based molecular techniques in 2013, described in more detail elsewhere [8].

In this study, an outbreak was defined as the occurrence of at least one reported confirmed rabies virus cattle death at one farm. For the analysis, three time series were constructed using information from the 100 rabies outbreaks that were reported in cattle herds of Costa Rica between January 1985 and December 2016.

The first time series was a monthly record of cattle deaths due to rabies virus infection. The second time series recorded the monthly number of outbreaks, while the third series had the monthly mortality by rabies virus infection. In this last time series, mortality was defined as the percent of deaths, due to rabies virus infection, in the farm where the outbreak was detected, i.e., for each farm was estimated as follows: (number of recorded deaths due to rabies * 100) / number of cattle heads present in the farm. When two or more outbreaks occurred in the same month, mortality was estimated by adding deaths and total cattle heads across all affected farms.

For 33 of the outbreaks, all before 2000, the number of cattle heads in farms where deaths by rabies were recorded needed to be imputed. This was done because spatially explicit data about cattle heads in Costa Rica are only available after 2013, the time when the agricultural holdings database (Sistema de Reconocimentos Ambientales, SIREA) was established [8, 43], and also to consider that cattle density has been reduced in the country. Therefore, under the valid assumption that neighbouring farms have similar herd sizes [39], a fully cross-validated regression tree was employed to estimate the missing herd sizes as function of farm coordinates and altitude. Briefly, the regression tree is a quantitative method where a set of rules are derived by looking at patterns of association between the independent variables and a response variable, having the ability to capture non-linear relationships between variables when compared with linear regression [44]. Full cross-validation means that parameters are estimated by fitting a set of models where, one at a time, each observation is left out of the model fitting and then that observation is predicted with parameters estimated from all other observations so that parameters are chosen by minimizing the difference between all predicted values and observations [45].

### Meteorological data

For the analysis, the definition of cold and hot ENSO phases from the National Oceanic and Atmospheric Administration (NOAA) was used [46]. This definition is based on the Oceanic Niño Index, the 3 month running average of temperature anomalies in the Niño 3.4 region (5^o^N-5^o^S, 120^o^-170^o^W), and where anomalies are based on 30 year base periods, which are updated every 7 years. The analyses employed monthly anomalies from the El Niño 4 index [47] which are based on measurements from the following area: 5^o^N-5^o^S, 160°E-150^o^W. This selection was based on previous research [32, 34, 49, 50] that has shown this index has the highest association with weather anomalies in Central America. For temperature and rainfall, gridded data, available at the Royal Netherlands Meteorological Institute (KNMI) climate explorer [51], were employed. Data were downloaded for the land surface contained in the area enclosed by the following coordinates: 11.00^o^N-8.55^o^N, 86.00^o^W-83.00^o^W, which roughly corresponds to Costa Rica and contains all the recorded outbreaks between 1985 and 2016. More specifically, data from the following two databases at KNMI were used for: (i) temperature: NOAA data from the Global Historical Climatology Network version 2 and the Climate Anomaly Monitoring System (GHCN_CAMS 2 m model), with a spatial resolution of 0.5° [52]; (ii) rainfall: data from the NOAA Climate Data Record (CDR) of Satellite-Gauge Precipitation from the Global Precipitation Climatology Project (GPCP), V2.3, with a spatial resolution of 2.5° [53].

### Statistical analysis

Cattle rabies outbreak and mortality cycles were studied with frequency domain time series methods, which are tools to study the cyclic behaviour of time series [54]. Periodograms, which show the distribution of power (i.e., variance) among different frequencies, i.e., the inverse of the period for a given cycle, were estimated by taking the Fourier transform of the time series. In a periodogram, a peak indicates a dominant frequency in the cyclic behaviour of a time series [54]. Periodograms were estimated for the annual time series rabies outbreaks and the El Niño 4 index, assuming that these two time series were stationary, i.e., with a constant mean and variance [54]. The seasonality of the epidemiological and meteorological data was assessed using a seasonal boxplot, i.e., a graph where boxplots for the 12 months in the year are sequentially drawn [54]. Meanwhile, the association between epidemiological and climatic time series was studied using cross wavelet coherence analysis. This is a time frequency time series analysis technique that can assess the non-stationary, i.e., changing through time, association between two time series. In this analysis, the association between cycles of different periods between two time series is studied through time, showing periods of time when the cycles are significantly coupled or not [48–50, 54–56].

Retrospective space-time cluster analyses were performed with the scan statistic [57]. Discrete poisson models [58] were implemented for the study period 1985–2016. For the analyses elliptic scanning windows were used. The elliptic window is the preferred choice when looking for clusters over a space that is asymmetric, i.e., like the map of Costa Rica where land occurs along a southeast to northwest axis, and the elliptic window has the advantage of converging to a circle when there is a cluster around a focal point, or an ellipse when clusters follow a pattern similar to a line [59]. The scan statistic was employed to search for high rates. Following the suggestion by Kulldorf et al. [57], the scan statistic was estimated with a scanning window whose maximum radius for the long axis of the ellipse generated an area covering half of the points where cattle rabies outbreaks were located. The number of reported rabies deaths and the population at risk for each outbreak were used in order to detect whether outbreaks were purely at random, or if clusters of high mortality existed. Four models were estimated: two models were set up with an annual aggregation of cases, and two with a monthly aggregation of cases. For the models of annual and monthly data aggregation, one model considered observations including the imputed values for the estimated populations with the regression trees, and one included only information for outbreaks where the population at risk had also been registered.

Finally, seasonal autoregressive (SAR) time series models were developed to study the linear association between the monthly number of outbreaks, the monthly mortality rates and the El Niño 4 index, temperature, and rainfall. The protocol to develop SAR models started by fitting “null models” that accounted for periodicities of the focal time series (outbreaks or mortality) by appropriately considering the correlation structure of the time series as inferred from the inspection of the auto-correlation function (ACF) and partial auto-correlation function (PACF) of the time series. The output of ACF is a plot that depicts the correlation of a time series with itself at different time lags, while the PACF output depicts a similar correlation but only considering consecutive time lags [54]. The resulting “null models” were then used to pre-whiten, also known as filtering, the time series of the climatic covariates. This process removes any common auto-correlation structure from the climatic time series; thus, when a cross-correlation function (CCF) is estimated from the residuals of the null model and the pre-whiten time series, no spurious association — emerging from the time series having a similar auto-correlation structure — is found. The CCF is a graphical representation of the correlation between two time series at different lags [54]. Following the inspection of the cross-correlation function, we built “full models” that considered all covariates at the significant lags (*p* < 0.05) with the highest correlations. These full models were then simplified by a process of backward elimination [60], where the model with the lowest Akaike Information Criterion (AIC) from a set of models with the same number of parameter was further simplified until a minimum AIC was reached [50]. The AIC is a trade-off function that considers the number of parameters and the likelihood of a model, allowing the selection of models based on AIC minimization [50]. To ease model interpretation, the mean of climatic time series were removed when fitting the SAR time series models [49], something that equals intercepts to the average value of the focal time series studied [54].

### Software

All geographic information systems procedures, mapping, regression trees, cross-wavelet analyses and the time series models were done with the statistical software R version 3.4.0. The scan cluster analyses were performed with SaTScan [57].

## Results

**Fig. 1:**
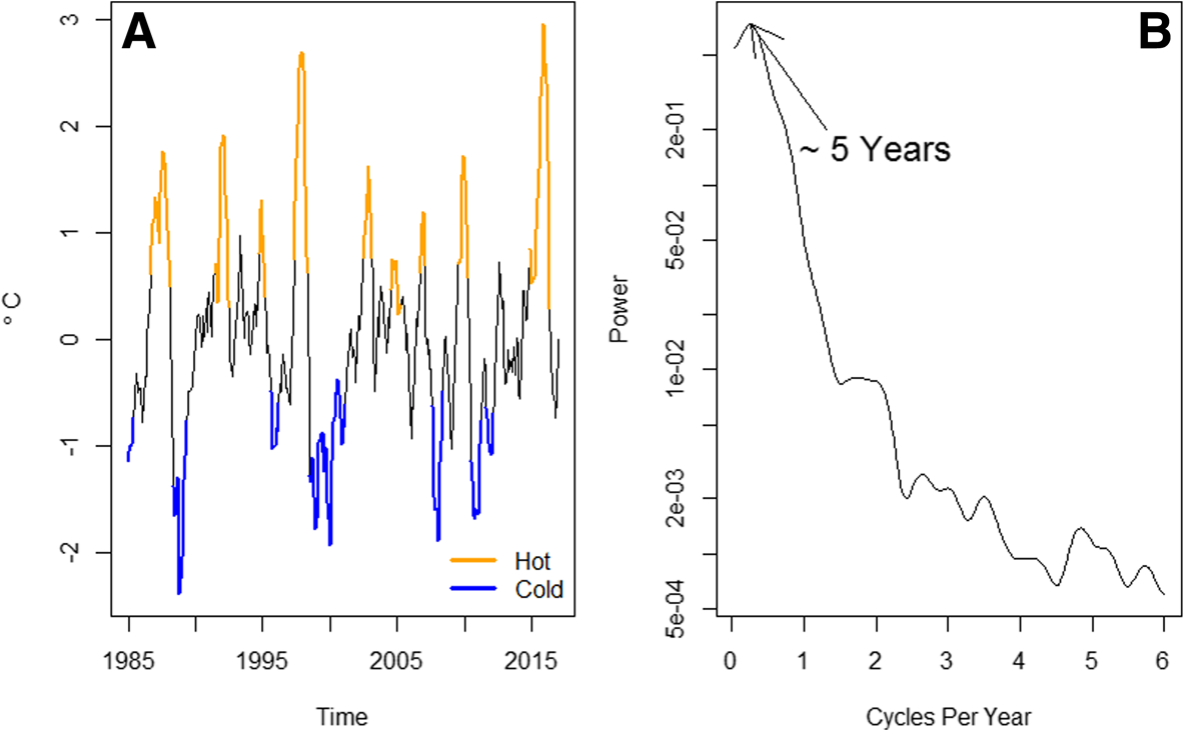
The El Niño Southern Oscillation (ENSO) dynamics (1985–2016). **a** Monthly time series of the El Niño 4 index from 1985 to 2016. ENSO events are highlighted; hot phases are shown in orange, cold phases in blue. **b** Periodogram for the monthly anomalies from the El Niño 4 index. The peak power value corresponds at 0.2 cycles per year, indicating that ENSO had an approximate oscillation period of 5 years over the study period

**Fig. 2:**
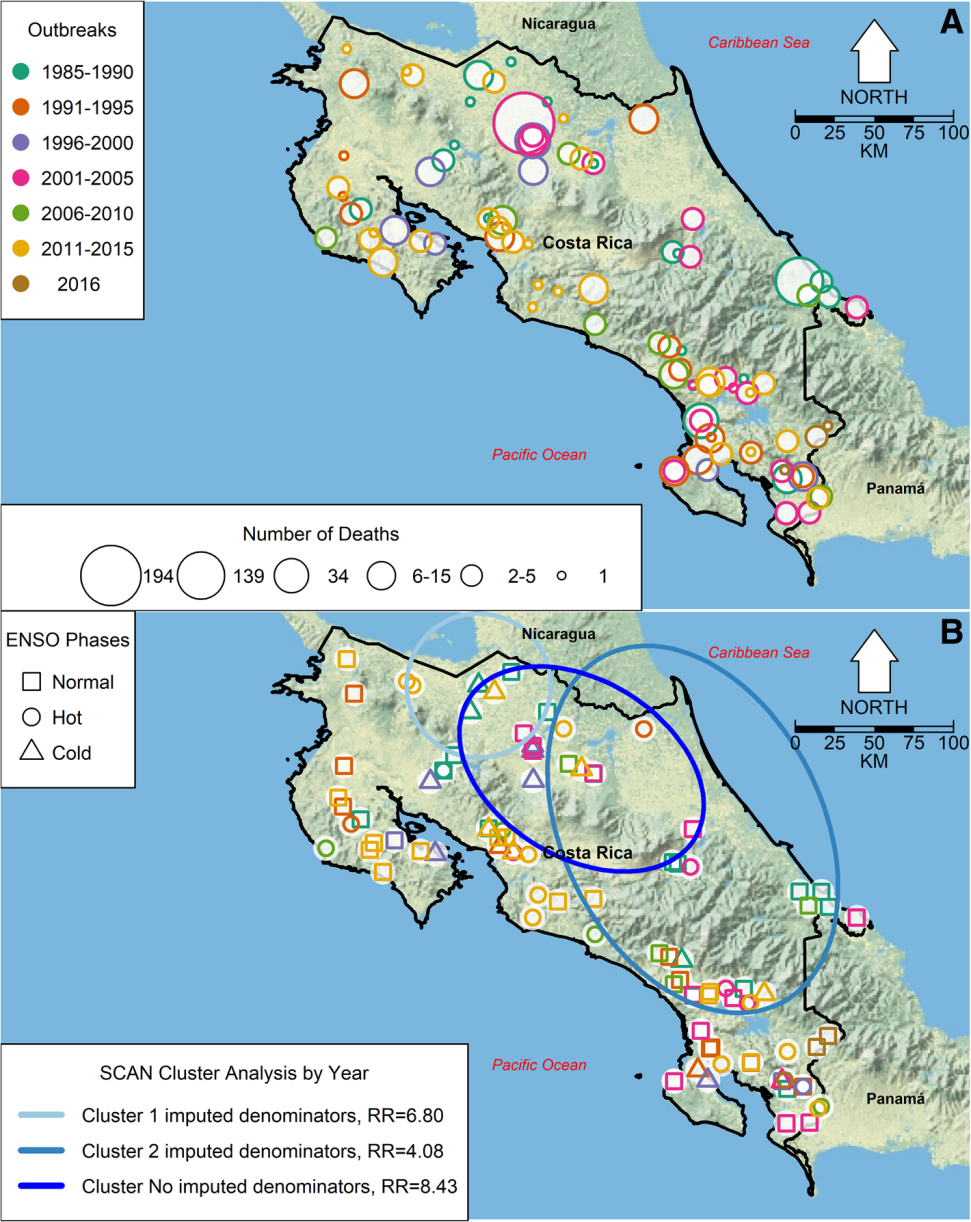
Geolocation of cattle rabies outbreaks in Costa Rica (1985–2016). **a** Map of Costa Rica with the period and size, i.e., number of rabies deaths, of cattle rabies outbreaks. Most outbreaks occurred between 2001 and 2005 and 2011–2015. Numbers of rabies deaths were grouped in 6 categories and most of the outbreaks had between 1 to 5 deaths. **b** Map of Costa Rica with the El Niño Southern Oscillation (ENSO) phase in which cattle rabies outbreaks occurred (square-normal, circle-hot and triangle-cold). Disease clusters from the annual SaTScan spatio-temporal analysis are indicated by circles. Clusters were estimated with and without imputed denominators. These maps were made using as base a public domain map from the US National Park Service [86]

During the study period (1985–2016), there were 9 El Niño events, the two biggest events, i.e., those accounting for the most extreme ENSO fluctuations, occurring in 1998 and 2015 (Fig. 1a). During this time period, corresponding to the peak power value of approximately 0.2 cycles per year, ENSO had an approximate oscillation period of 5 years, as suggested by a periodogram of the monthly anomalies from the El Niño 4 index (Fig. 1b). During the same time period, rabies outbreaks in cattle occurred all over Costa Rica (Fig. 2a). As shown in Fig. 2a, most of the outbreaks had between 1 and 5 rabies deaths, but there were two large outbreaks of 139 and 194 deaths in 1985 and 2003, respectively (see Additional file 1: Figure S1A), which occurred in the Atlantic basin of the country. Figure 2a shows that outbreaks were very common between 1986 and 1991, 2001 and 2005 as well as between 2011 and 2015. Figure 2a also shows that most outbreaks occurred in the lowlands of Costa Rica. Additional file 2: Figure S2 is a video showing the month, year and size of each individual outbreak and ENSO phase. Further inspection of cattle rabies death records suggests that most deaths have occurred in April and July (Additional file 1: Figure S1B), which have been associated with the large outbreaks of 1985 and 2003 (Additional file 1: Figure S1A and C). Nevertheless, there is no seasonality in the number of rabies deaths recorded, based on the median values (zero) of the boxplots for cattle mortality in the whole territory of Costa Rica (Additional file 1: Figure S1D). Indeed, it seems that rabies deaths randomly happen throughout the year.

Figure 2b shows results from the annual scan spatio-temporal cluster analysis about rabies mortality in cattle. Two datasets were considered, a dataset for which denominators, i.e., cattle herd size were all known, and a dataset for which some denominators were imputed. In the two analyses, clusters of cattle rabies deaths were identified in northeastern Costa Rica, in the Atlantic basin. There was an important spatial overlap between the only cluster identified with the dataset without imputed denominators (which only spanned 2003) and Cluster 1 from the analysis with imputed denominators (which spanned from 1985 to 1999). Cluster 2 coincided temporally (from 2003 to 2015) and to a lesser extent spatially with the cluster from the analysis without imputed data. Similar results were observed when the cluster analysis was performed for monthly data of cattle rabies deaths (Additional file 3: Figure S3), where the 2003 outbreak of 194 dead cows was identified as an important cluster independently of considering the imputed denominators, and clusters were also mainly located in the Atlantic basin of Costa Rica. The calculated relative risks, where relative risk is an adimensional proportion defined as the ratio between the probability of cattle death by rabies happening inside the spatial cluster when compared with the probability of such event outside the cluster area, reflect the increased risks for cattle deaths by rabies virus infections in these identified areas when compared with the surrounding landscape.

**Fig. 3:**
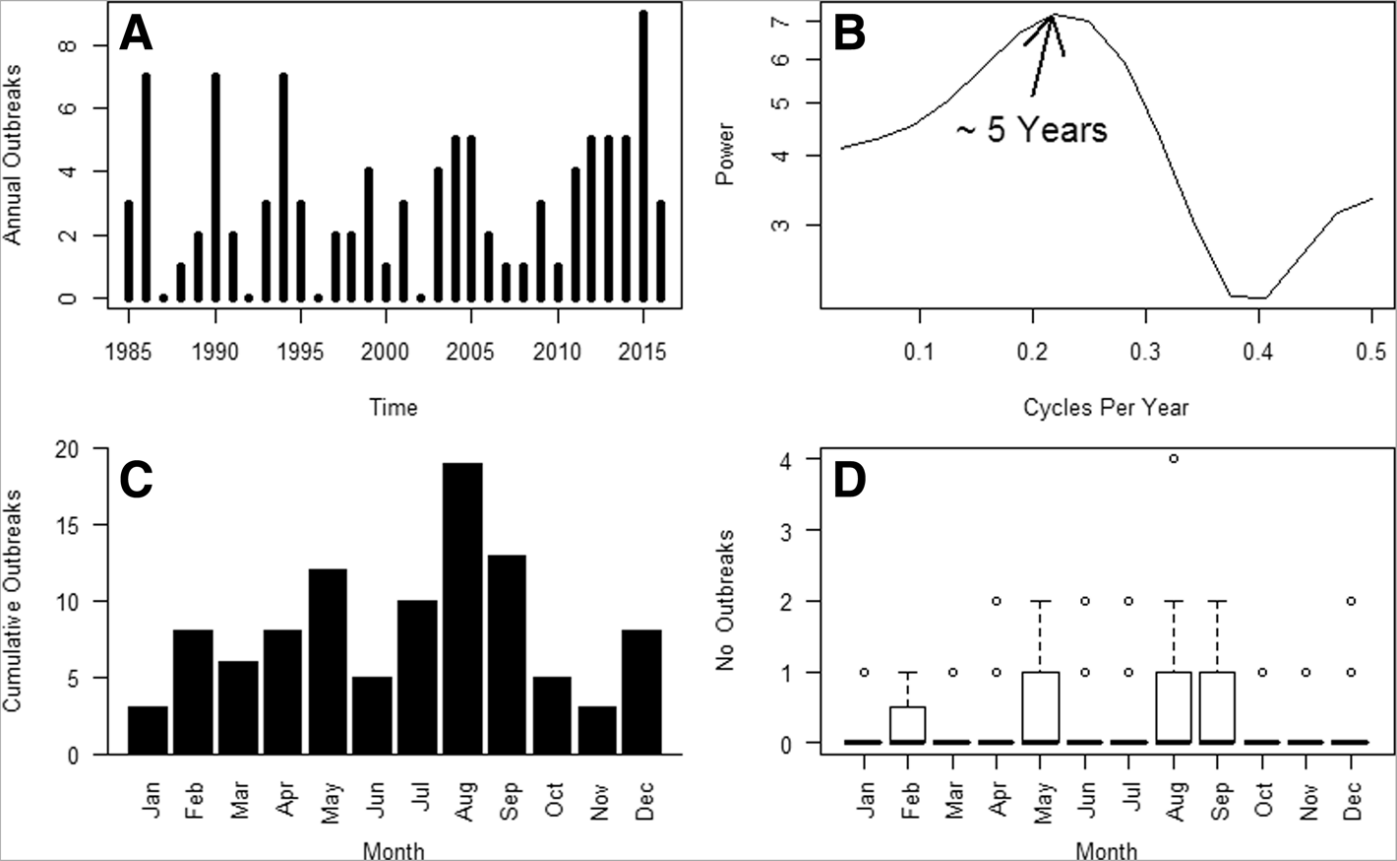
Temporal patterns of cattle rabies outbreak numbers in Costa Rica (1985–2016). **a** Annual time series of outbreak numbers in Costa Rica from 1985 to 2016. **b** Periodogram of the annual time series of outbreak numbers in Costa Rica from 1985 to 2016. The peak power value corresponds at 0.2 cycles per year, indicating that cattle rabies outbreak numbers had an approximate oscillation period of 5 years over the study period. **c** Barchart of the cumulative number of rabies outbreaks per month during the study period. The highest number of rabies outbreaks occurred in August, September and May. **d** Boxplots of monthly rabies outbreak numbers. For all months, from January to December, the median value for all monthly boxplots was 0, which suggests that there is no seasonality in the number of monthly cattle rabies outbreaks

The incidence of outbreaks, independently of the number of rabies deaths, was variable across the study period, with one to nine outbreaks per year occurring during the studied years, and with cyclical fadeouts every few years up to 2002 (Fig. 3a).

There is a periodicity of approximately 5 years in the number of annual outbreaks as suggested by the annual outbreak number time series periodogram (Fig. 3b), the same oscillation period observed for ENSO. Outbreaks occurred more frequently in the months of August, September and May (Fig. 3c), and this seems to be an incipient seasonal pattern, yet at any given month no outbreaks regularly happened (Fig. 3d).

**Fig. 4:**
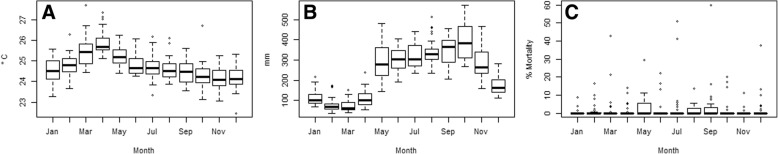
Weather and cattle rabies mortality seasonal patterns in Costa Rica. **a** Monthly temperature (°C) boxplots. **b** Monthly rainfall (mm) boxplots. **c** Monthly cattle rabies mortality (%) boxplots. All boxplots are based on data from January 1985 to December 2016

The lack of seasonality in cattle rabies deaths, and outbreaks is in sharp contrast with the seasonal weather of Costa Rica. Temperature (Fig. 4a) and rainfall (Fig. 4b) show a clear unimodal pattern, with two seasons. Temperature reaches a maximum in April and is lowest in December (Fig. 4a). For rainfall, there is a dry season between December and April, followed by a wet season the rest of the year (Fig. 4b). Nevertheless, mortality associated with cattle rabies outbreaks (Fig. 4c), although proportionally higher in May, August and September in specific years, is not regularly higher in any given month of the year.

**Fig. 5:**
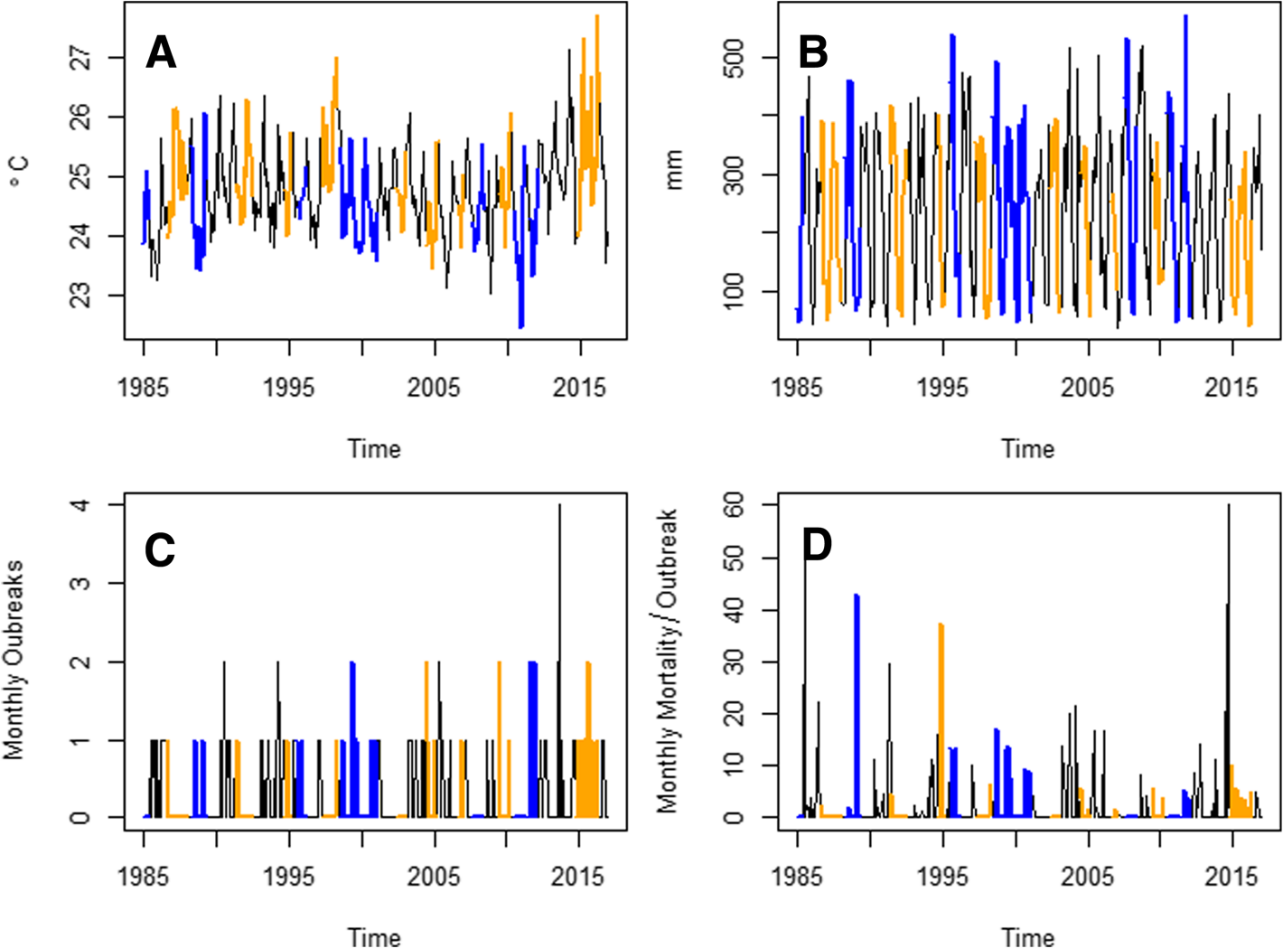
The El Niño Southern Oscillation (ENSO) events and monthly weather and cattle rabies time series (1985–2016). **a** Temperature (°C). **b** Rainfall (mm). **c** Cattle rabies outbreak numbers. **d** Cattle rabies mortality (%). ENSO events are highlighted, hot phases are shown in orange, cold phases in blue, and normal months in black

The signature of ENSO in temperature (Fig. 5a) suggests that the hot ENSO phase is associated with higher than average temperatures in Costa Rica. Meanwhile, the cold phase of ENSO is associated with increased rainfall in Costa Rica (Fig. 5b). After 1998, outbreaks of cattle rabies seemed to be more prone to occur during the hot and cold phases of ENSO (Fig. 5c), and some cattle rabies outbreaks with high mortality rates occurred during the cold phase of ENSO (Fig. 5d).

**Fig. 6:**
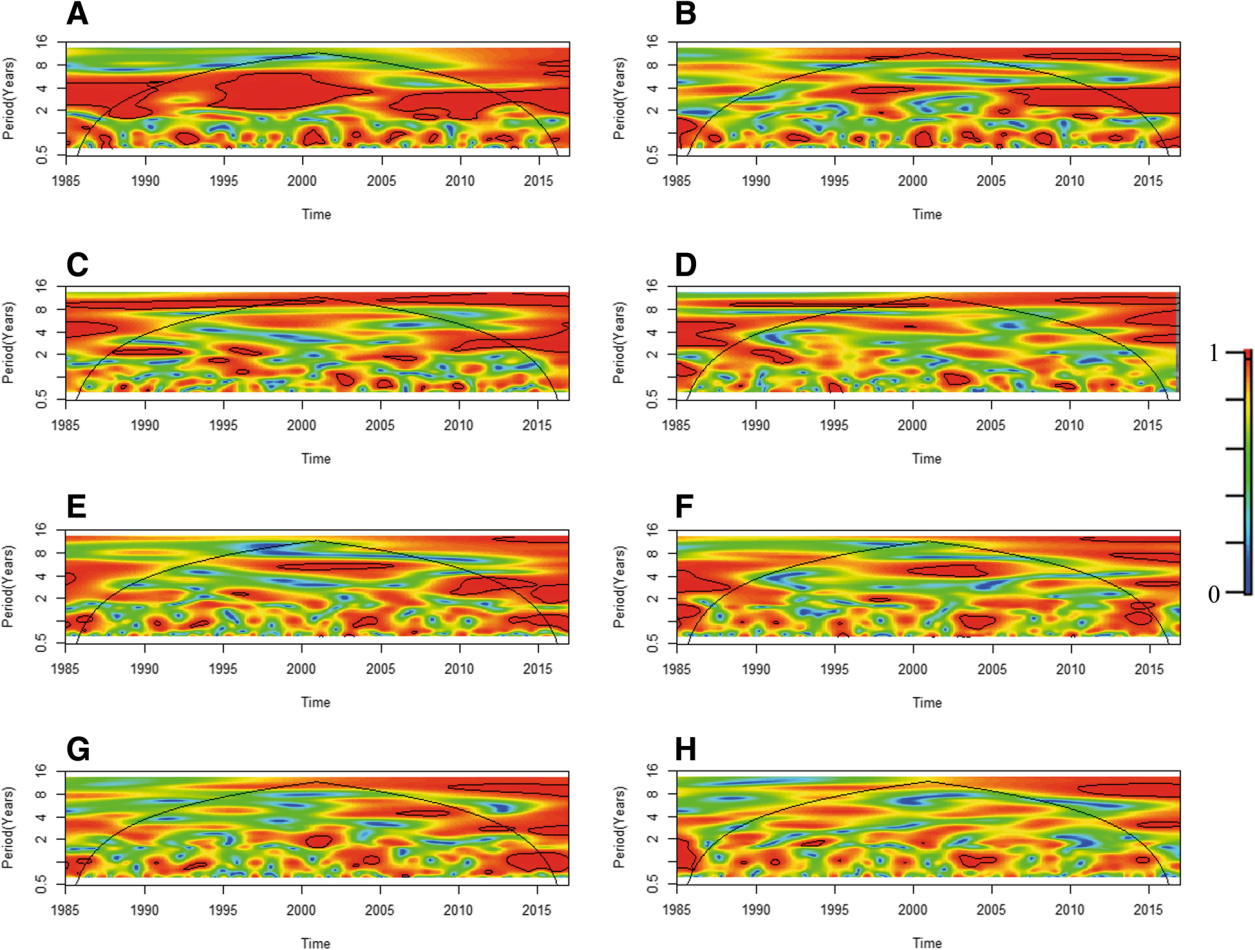
Cross wavelet coherence analysis for monthly time series (1985–2016). Panels show the cross-wavelet coherence between: **(a)** The El Niño Southern Oscillation (ENSO) and temperature. **b** ENSO and rainfall. **c** ENSO and number of cattle rabies outbreaks. **d** ENSO and cattle rabies outbreak mortality. **e** Temperature and cattle rabies outbreaks. **f** Temperature and cattle rabies outbreak mortality. **g** Rainfall and cattle rabies outbreaks **(h)** Rainfall and cattle rabies outbreak mortality. A cross wavelet coherence scale is presented at the right side of the figure, which goes from zero (blue) to one (red). Red regions in the plots indicate frequencies and times for which the two series share power (i.e., variability). The cone of influence (within which results are not influenced by the edges of the data) and the significant coherent time-frequency regions (*p* < 0.05) are indicated by black solid lines

The cross wavelet coherence analysis suggests that interannual cycles of temperature and ENSO are highly coherent at periods of 2–5 years over the studied period (Fig. 6a). For rainfall (Fig. 6b), cycles of 3–6 years of ENSO and rainfall were highly coherent around the 1998 ENSO event, and have become increasingly coherent for the same periods (2–4 years) since 2006. Cattle rabies outbreaks have been coherent at larger time scales, 8–10 years, with ENSO (Fig. 6c), and cycles have become increasingly associated at scales of 2–4 years after 2010. Monthly cattle rabies mortality shows no major significant association with ENSO during the studied period (Fig. 6d). The cross wavelet coherence analysis suggests that number of outbreaks (Fig. 6e) and cattle rabies mortality (Fig. 6f) between 1998 and 2005, for interannual cycles of periods of 4–6 years, were highly associated with temperature. Moreover, for outbreak numbers, the association was also significant after 2010 for cycles of periods of 2–4 years (Fig. 6e). Rainfall was associated with number of outbreaks (Fig. 6g) for cycles of periods of 4–6 years from 2006 to 2008. Meanwhile, there was no major significant association between cycles in rainfall and mortality (Fig. 6h) during the study period.

**Fig. 7:**
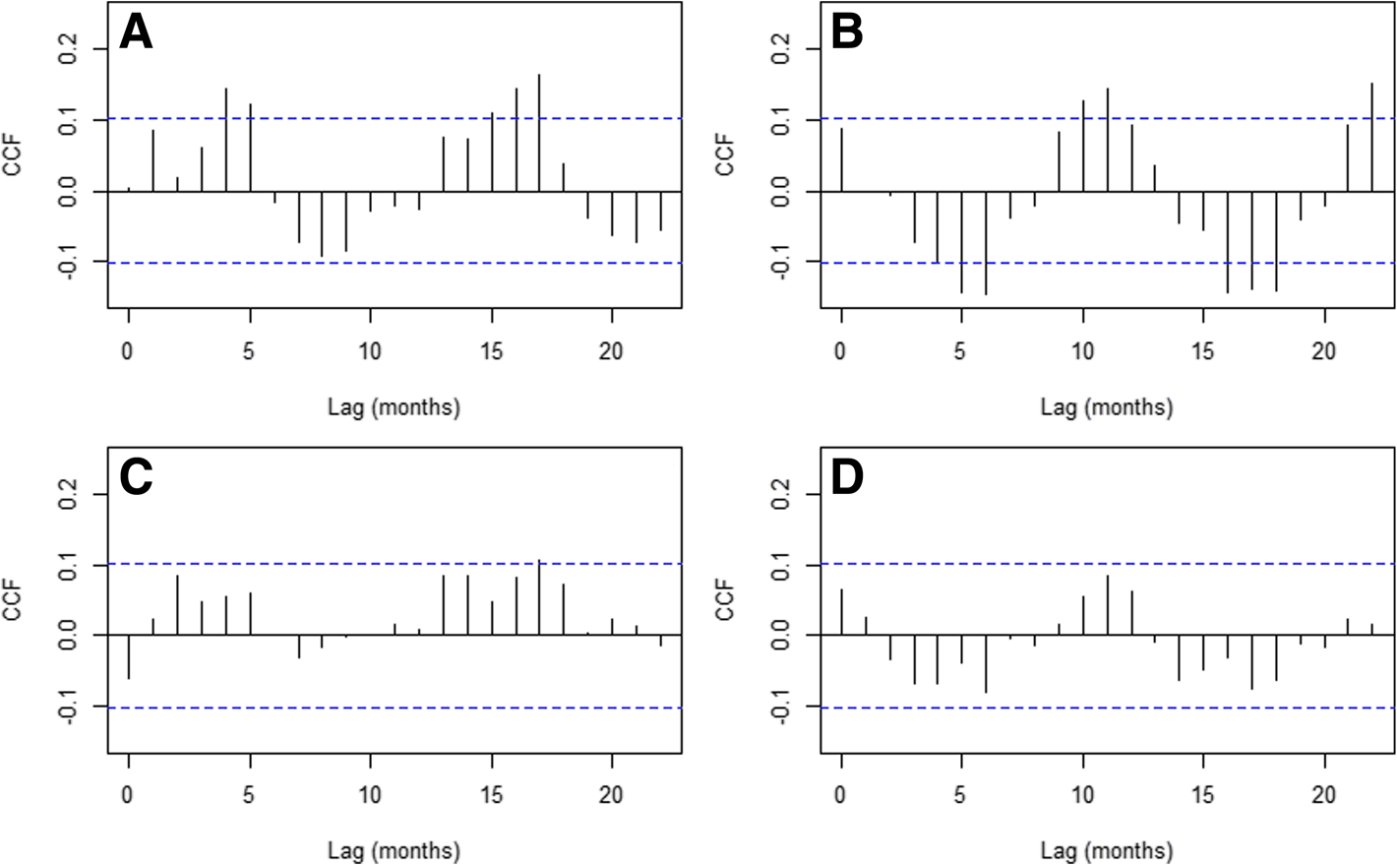
Cross correlation functions between weather variables and cattle rabies. **a** Temperature and cattle rabies outbreaks (most significant correlations at lags 4 and 17 months). **b** Rainfall and cattle rabies outbreaks (most significant correlations at lags 6, 11 and 16 months). **c** Temperature and cattle rabies outbreak mortality (significant at lag 17 months). **d** Rainfall and cattle rabies outbreak mortality (no significant lag). Blue dashed lines indicate the 95% confidence limits for correlations that can be expected by random

The number of outbreaks time series was not strongly autocorrelated, i.e., there was no clear pattern of significantly (*p* < 0.05) decreasing auto-correlation with time lag (Additional file 4: Figure S4A), and showed periodicities every 2 months (Additional file 4: Figure S4B), which was considered when fitting a null SAR time series model (Additional file 5: Table S1). Moreover this time series had no significant linear association with ENSO 4 over the studied period and up to 22 months of lag (Additional file 4: Figure S4C). A similar pattern of low auto-correlation was observed for the outbreak mortality time series (Additional file 4: Figure S4D), which showed periodicities of 2 months (Additional file 4: Figure S4E), also considered when fitting a null SAR time series model (Additional file 6: Table S2). The mortality time series also had no significant association with ENSO 4 up to 22 months of lag (Additional file 4: Figure S4F). When considering covariates to develop a full model for the number of outbreaks time series (Additional file 5: Table S1), lags 4 and 17 of temperature were considered (Fig. 7a) and lags 6, 11 and 16 of rainfall (Fig. 7b). Meanwhile, the full model for the mortality rate only considered lag 17 of temperature as covariate (Fig. 7c), since no significant (*p* < 0.05) association was found with rainfall (Fig. 7d).

The best time series model for the number of outbreaks (Table 1) had temperature with 17 months of lag and rainfall with 16 months of lag as covariate, having a positive coefficient for temperature and a negative one for rainfall. For cattle rabies mortality, the best model considered temperature with 17 months of lag as covariate (Table 2) with a positive coefficient. These results indicate that outbreaks and mortality are more likely to increase following hot-spells. Outbreaks are also less prone if the hot spell is followed by rain in an amount that offsets the positive impact of hot temperatures from the previous time lag (Table 1).

**Table 1:** Parameter estimates for the best time series model of monthly cattle rabies outbreak numbers (1985–2016). The first column shows the estimated parameters and time lag (in months). The second column depicts the estimates. Covariates for the best model included temperature (with 17 months of lag), and rainfall (with 16 months of lag)

Parameter (Lag)	Estimate ± S.E.
Intercept	−0.069 ± 0.097
Φ SAR (3)	0.1403 ± 0.0506
Temp (17)	0.1301 ± 0.0365
Rain (16)	−0.0006 ± 0.0002

*SAR* Seasonal autoregressive, *Temp* Temperature, *S.E.* Standard Error

**Table 2:** Parameter estimates for the best time series model of monthly cattle rabies mortality (1985–2016). The first column shows the estimated parameters and time lag (in months). The second column depicts the estimates. Covariates for the best model included temperature (with 17 months of lag)

Parameter (Lag)	Estimate ± S.E.
Intercept	−0.0608 ± 1.2243
Φ SAR (2)	0.1186 ± 0.0506
Temp (17)	1.0421 ± 0.4621

*SAR* Seasonal autoregressive, *Temp* Temperature, *S.E.* Standard Error

## Discussion

The similar period, around 5 years, of interannual oscillations in ENSO and cattle rabies outbreaks is suggestive of an association between changes in the occurrence of rabies outbreaks and ENSO phases. This type of association has been widely found for several diseases, e.g., malaria [32], dengue [61], cutaneous leishmaniasis [34, 49] in Central America, but also elsewhere, e.g. Asia [62], Africa [29, 63, 64] and Oceania [65]. Although this formal analysis did not find signatures of ENSO on outbreak occurrence and cattle mortality dynamics, these two time series were associated with temperature and rainfall (outbreak occurrence only). As further shown by the analysis, the dynamics of temperature and rainfall in the studied area had interannual cycles associated with ENSO, thus implying the observed dynamics might be sensitive to ENSO. In the studied data, no seasonality in the number of monthly rabies deaths and recorded outbreaks was observed.

Clusters identified with the scan statistic were mainly located in the Atlantic basin of Costa Rica, something suggested in a preceding description of most of the studied dataset [8]. The largest outbreaks and high mortality clusters occurred in the Atlantic basin of Costa Rica, a fact that might be related with common vampire bat ease of movement or dispersal, in an area slightly more humid [25] than the Pacific basin of Costa Rica [66]. Similarly, cattle rabies geolocation patterns might reflect common vampire bat abundance patterns, which are known to be associated with specific environments, defined by the niche of bats [13, 67, 68], and where vampire abundance could increase rabies virus transmission [14, 69]. An additional reason could be that surveillance and prevention might be less effective in the Atlantic basin of Costa Rica. For example, underreporting could delay appropriate action and promote a reduced coverage of cattle rabies vaccination, which in turn might lead to larger outbreaks, given an increased susceptibility in host populations without rabies vaccination, as predicted by mathematical models dealing with risk perception and infectious disease transmission prevention [70]. Actually, the large 1985 outbreak occurred when no control programme was in operation. In 2003, high cattle mortality was likely fuelled by late reports to the veterinary authority, as well as, a difficult access to the study area, a situation that was exacerbated by heavy rainfall which delayed, by at least a month, cattle vaccination. We suspect common vampire bats were exceptionally abundant, and likely linked with a high rabies virus prevalence. The fact that the only rabies virus isolation from a common vampire bat in Costa Rica was made where the 2003 outbreak occurred might support this further, but pure coincidence can not be ruled out as frequency of bat testing for rabies virus is not available.

This analysis indicated that temperature might have a positive impact on the transmission of rabies virus to cattle, as suggested by the positive association of temperature with outbreak occurrence and cattle rabies mortality. The lag of this association roughly corresponds to two common vampire bat generations (16 to 17 months), and is in accordance with observations about increases in survival and recruitment of common vampire bats following hot temperatures [25]. Specifically, common vampire bat pre-weaning time is around 3 months, a period when vampire pups mainly feed on milk [71]. Suckling vampire pups are more likely to survive at higher temperatures given the increased blood foraging in hotter/dryer conditions by adult common vampire bats [26]. Here, it is important to note that food deprivation for two to 3 days (e.g. through adverse weather conditions such as strong rains) might become fatal for young bats [72], which also have a high failure rate to obtain blood meals on their own [73]. Therefore, any change that increases the survival of young vampire pups might be critical to increase rabies virus transmission by increasing the size of common vampire bat populations. Pre-weaning vampire pups also start to receive regurgitated blood during their first 3 months of age, a key event for rabies virus transmission within common vampire bats [71, 74]. Indeed, food deprivation in common vampire bats can be compensated through reciprocal blood sharing by roost mates [73]. Thus, a likely increase in vampire pup survival during the pre-weaning period, followed by the six to 7 months common vampire bats need to reach adulthood, when bats are between 9 and 10 months old, and also become sexually mature, with a diet exclusively based on fresh blood, could lead to a larger cohort of reproducing common vampire bats [14, 75]. This larger cohort then takes seven additional months (thus adding up to around 16 to 17 months since birth) for the gestation of a new *D. rotondus* vampire cohort [76] which will require additional blood as they try to raise their pups. Most likely this will increase blood foraging pressure, thus, rendering plausible an increase in rabies virus transmission 16–17 months after hotter temperatures might have promoted breeding and increased the survival of pre-weaning vampire pups.

Meanwhile, a mechanism where the delays are due to changes in the rabies virus incubation period in cattle or common vampire bats is unlikely, provided experimental studies have shown incubation periods in the order of one to 2 months in cattle [77], and two to 4 weeks in bats [78]. Nevertheless, rabies virus incubation period is known to be highly variable depending upon inoculation site [78] and viral loads [79]. The variability in rabies virus incubation period could be one of the reasons behind the lack of a seasonality in cattle rabies outbreaks, although the occurrence of large outbreaks where the onset of clinical signs is more or less synchronous suggests that, at least in outbreak foci, cattle have a homogenous incubation period [80]. The occurrence time for the two largest cattle rabies outbreaks, which happened during April and July, and the frequent occurrence of outbreaks during the months of August, September and May, further suggest that common vampire bat ecology plays a major role explaining the lagged effects of temperature on cattle rabies outbreaks. For example, Turner [28] found that most bats were pregnant or lactating in the months of April–May and July–October, an observation coinciding with records from Costa Rica [26], suggesting increased transmission might be related to maternal care of pre-weaning common vampire bats, a possibility supported by the increased rabies virus exposure of juvenile and subadult common vampire bats, which have shown higher rabies virus seroprevalence than adults [14].

In contrast to the positive impacts of temperature on outbreak occurrence and size, this analysis showed that rainfall had a negative impact on cattle rabies outbreak frequency in the SAR time series models. This negative association might emerge from rainfall impacts on bat foraging. For example, a study found that rainfall increased flight metabolism, while reducing or ceasing foraging activities [81]. Reductions in vampire foraging time could then diminish the exposure of cattle to rabies virus infected common vampire bats. Similarly, the location of cattle by common vampire bats could become more difficult following rainy periods, when a reduction in cattle surface body temperature could interfere with common vampire bat thermoreceptors used to locate their prey [82]. Furthermore, abundant rain after a hot spell might diminish vampire fecundity, via a reduction in common vampire bat movement and mating between roosts [24]. For example, a study in Costa Rica showed that common vampire bats stayed away from their roost more time during the dry than the wet season [26].

Limitations of this study include the following: (i) The mortality analysis considered estimated denominators in a fraction of the data. Nevertheless, as shown by the scan cluster analysis, the results were robust when those “estimated” denominators were ignored. (ii) Data on cattle mortality were less robust compared to outbreak data which may hide the true number of rabies cases. (iii) The impact of rabies vaccination on cattle mortality patterns was not assessed due to lacking systematic information about vaccination rates in affected herds. (iv) It was assumed that rabies virus was only transmitted by common vampire bats [12, 20]. However, it is possible that other wildlife species might have been involved in rabies virus transmission, as have been documented for raccoons, skunks and foxes [5, 76, 83–85]. In Costa Rica, a cattle rabies case was associated with the rabies virus strain of *Tadarida brasiliensis*, an insectivorous bat species [8]. In that sense, sequencing rabies viruses isolated in cattle rabies outbreaks might help to identify other species potentially involved in rabies virus transmission. Phylogenetic analyses could also help to better understand geographical and temporal structures of outbreaks, and whether common vampire bats and other wildlife host species are involved in local enzootic cycles or epizootic waves [73, 83]. Further efforts are also necessary to understand rabies virus spillovers across species and the role that bat immunity has on the persistence of rabies virus among common vampire bats under changing environments, to fully untangle the impacts that changing rainfall and temperature patterns have on common vampire bat population structure and dynamics, as well as, rabies virus transmission.

Finally, the results of this study clearly indicate that the occurrence, and mortality of cattle rabies outbreaks are associated with weather fluctuations in Costa Rica. Further eco-epidemiological field studies are necessary to articulate the mechanisms behind the associations observed in this study, especially regarding the hypothesis that outbreaks, and their magnitude, might be driven by changes in the population dynamics of vampire bats triggered by ENSO phases. The resulting information from those studies will be useful not only to understand rabies virus transmission ecology, but also to propose appropriate intervention measures to control this major veterinary public health problem.

## Conclusions

Further efforts are necessary to articulate the mechanisms underpinning the association between weather changes and cattle rabies outbreaks. One hypothesis is that exacerbation of cattle rabies outbreaks might be mediated by impacts of weather conditions on common vampire bat movement and access to food resources on its natural habitats. Further eco-epidemiological field studies could help to understand rabies virus transmission ecology, and to propose sound interventions to control this major veterinary public health problem.

## Additional files

Additional file 1: **Figure S1.** Temporal patterns of cattle rabies outbreak mortality in Costa Rica (1985–2016). (A) Annual time series of cattle deaths from 1985 to 2016. Peak values occurred in 1985 and 2003, with 149 and 193 cattle deaths, respectively. (B) Bar chart of monthly cumulative cattle deaths from 1985-2016. The highest number of deaths occurred in April and July. (C) Monthly time series of cattle deaths from 1985 to 2016. It reflects both outbreaks during a year and cattle rabies deaths per month. Peak values occurred in 1985 and 2003. Note that in year 1995, although there is no peak value of cattle rabies death, there were many small outbreaks. (D) Boxplots of monthly cattle rabies deaths from 1985 to 2016. For all months from January to December, the median value of the boxplots is 0, which suggests that there is no seasonality in monthly cattle rabies deaths. (PDF 19 kb)

Additional file 2: **Figure S2.** Video showing the geolocation and size of cattle rabies outbreaks (1985-2016). In the video the current outbreak is shown in red while past outbreaks are in blue. Circles are proportional to the number of cattle rabies deaths (for guidance see the inset legend at the bottom of the map) and for every outbreak, the month and year, the exact number of cattle rabies deaths, and the El Niño Southern Oscillation (ENSO) phase are indicated in the upper left corner of the map. (MP4 48445 kb)

Additional file 3: **Figure S3.** Monthly scan cluster analysis. Cluster 1 (circle with dash line in green color) and cluster 2 (circle with dash line in red color) were calculated with data for which denominators were imputed; cluster 3 (circle with dash line in purple color) and cluster 4 (circle with dash line in pink color) were calculated with no imputed denominators. Relative risks (RR) reflect the magnitude of the difference of the deaths in the cluster to regular deaths, which is the expected death assuming they occurred as driven by a homogenous Poisson process. This figure was made using a public domain map from the US National Park Service (https://www.nps.gov/hfc/carto/data-sources.cfm) as background. (PDF 97 kb)

Additional file 4: **Figure S4.** Correlation functions. (A) Auto-correlation function (ACF) of the monthly cattle rabies outbreak number time series; it shows the data were not strongly auto-correlated. (B) Partial auto-correlation function (PACF) of the monthly cattle rabies outbreaks time series data shows that there is a periodicity of 3 months. (C) Cross-correlation function (CCF) of the monthly cattle rabies outbreak number time series data with the ENSO4 index data. This CCF shows no significant linear association over the studied period, when considering up to 22 months of lag. (D) ACF of the monthly outbreak mortality time series. This time series shows low autocorrelation. (E) PACF of the monthly outbreak mortality time series. This time series shows a periodicity of 2 months. (F) CCF of the monthly outbreak mortality time series data with the ENSO4 index data. This CCF shows no significant linear association over the studied period, when considering up to 22 months of lag. In all panels blue dashed lines indicate the 95% confidence limits for correlations that can be expected by random. (PDF 17 kb)

Additional file 5: **Table S1.** Selection of the best monthly cattle rabies outbreaks time series model. Columns indicate the type of model (models): full or the backward elimination round. The Akaike Information Criterion (AIC) is a model selection criterion which is minimized by the best model. The AIC for the best models of each selection round are bolded. o and x indicate, respectively, the presence or absence of a variable in a model. Temp is an abbreviation for temperature. Time lags are in months. (PDF 187 kb)

Additional file 6: **Table S2.** Selection of the best monthly cattle mortality time series model. Columns indicate the type of model: Null, or full model. The Akaike Information Criterion (AIC) is a model selection criterion which is minimized by the best model. The best model has its AIC bolded. o and x indicate, respectively, the presence or absence of a variable in a model. Temp is an abbreviation for temperature. Time lags are in months. (PDF 174 kb)

## Abbreviations

ACF: Auto-correlation function
AIC: Akaike Information Criterion
AR: Autoregressive
CCF: Cross-correlation function
CDR: Climate Data Record
*D.*: *Desmodus*
DNA: Deoxyribonucleic acid
ENSO: El Niño Southern Oscillation
GHCN_CAMS 2 m model: Global Historical Climatology Network version 2 and the Climate Anomaly Monitoring System
GPCP: Global Precipitation Climatology Project
GPS: Global Positioning System
KNMI: Royal Netherlands Meteorological Institute
PACF: Partial auto-correlation function
RNA: Ribonucleic acid
RR: Relative risks
S.E.: Standard error
SAR: Seasonal autoregressive
SaTScan: Software for spatial, temporal, or space-time scan statistics
SENASA: Servicio Nacional de Salud Animal
SIREA: Sistema de Reconocimentos Ambientales
Temp: Temperature
US: United States
