# Supplementary material for: Assessing changing weather and the El Niño Southern Oscillation impacts on cattle rabies outbreaks and mortality in Costa Rica (1985–2016)
Source: BMC Vet Res. 2018 Sep 17;14:285. doi: 10.1186/s12917-018-1588-8 (PMC6142330; doi:10.1186/s12917-018-1588-8)

Supplementary Figure S1 Temporal patterns of cattle rabies outbreak mortality in Costa Rica (1985-2016). **(A)** Annual time series of cattle deaths from 1985 to 2016. Peak values occurred in 1985 and 2003, with 149 and 193 cattle deaths, respectively. **(B)** Bar chart of monthly cumulative cattle deaths from 1985-2016. The highest number of deaths occurred in April and July. **(C)** Monthly time series of cattle deaths from 1985 to 2016. It reflects both outbreaks during a year and cattle rabies deaths per month. Peak values occurred in 1985 and 2003. Note that in year 1995, although there is no peak value of cattle rabies death, there were many small outbreaks. **(D)** Boxplots of monthly cattle rabies deaths from 1985 to 2016. For all months from January to December, the median value of the boxplots is 0, which suggests that there is no seasonality in monthly cattle rabies deaths.

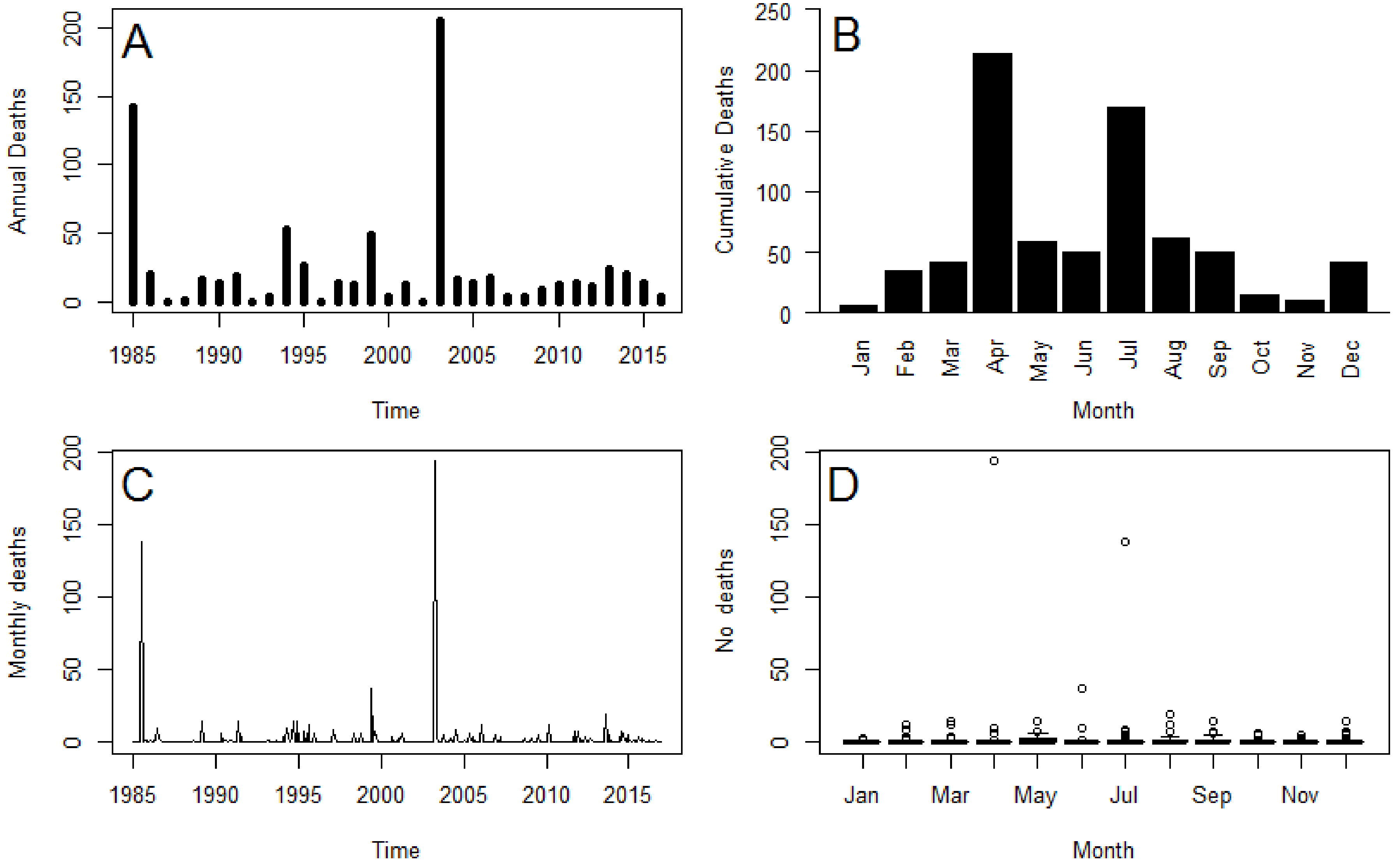

Supplement: Supplementary file 1 — Figure S1. Temporal patterns of cattle rabies outbreak mortality in Costa Rica (1985–2016). (A) Annual time series of cattle deaths from 1985 to 2016. Peak values occurred in 1985 and 2003, with 149 and 193 cattle deaths, respectively. (B) Bar chart of monthly cumulative cattle deaths from 1985-2016. The highest number of deaths occurred in April and July. (C) Monthly time series of cattle deaths from 1985 to 2016. It reflects both outbreaks during a year and cattle rabies deaths per month. Peak values occurred in 1985 and 2003. Note that in year 1995, although there is no peak value of cattle rabies death, there were many small outbreaks. (D) Boxplots of monthly cattle rabies deaths from 1985 to 2016. For all months from January to December, the median value of the boxplots is 0, which suggests that there is no seasonality in monthly cattle rabies deaths. (PDF 19 kb) [file 12917_2018_1588_MOESM1_ESM.pdf]
