# Supplementary material for: Assessing changing weather and the El Niño Southern Oscillation impacts on cattle rabies outbreaks and mortality in Costa Rica (1985–2016)
Source: BMC Vet Res. 2018 Sep 17;14:285. doi: 10.1186/s12917-018-1588-8 (PMC6142330; doi:10.1186/s12917-018-1588-8)

Supplementary Figure S3 Monthly scan cluster analysis. Cluster 1 (circle with dash line in green color) and cluster 2 (circle with dash line in red color) were calculated with data for which denominators were imputed; cluster 3 (circle with dash line in purple color) and cluster 4 (circle with dash line in pink color) were calculated with no imputed denominators. Relative risks (RR) reflect the magnitude of the difference of the deaths in the cluster to regular deaths, which is the expected death assuming they occurred as driven by a homogenous Poisson process. This figure was made using a public domain map from the US National Park Service (https://www.nps.gov/hfc/carto/data-sources.cfm) as background.

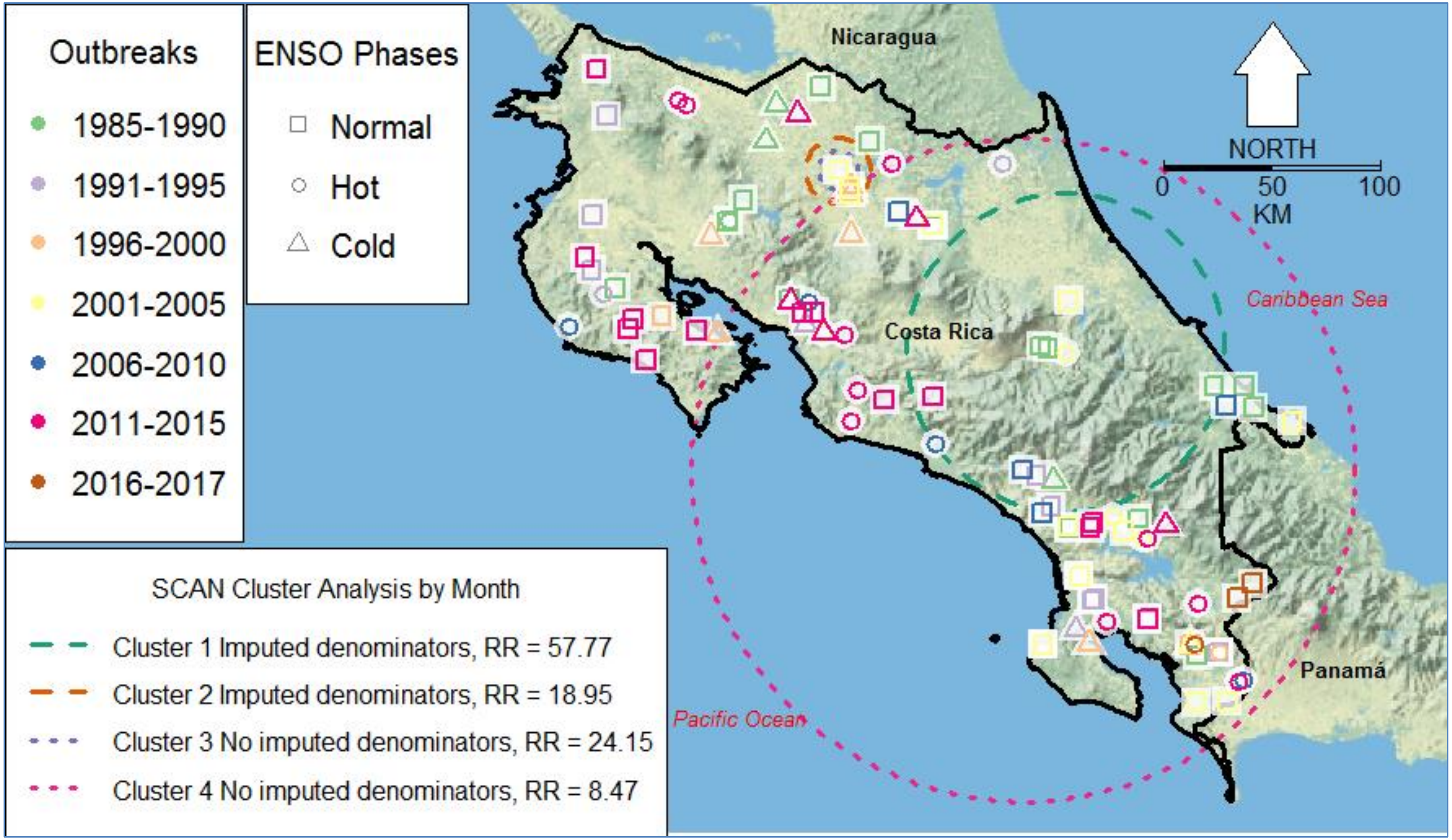

Supplement: Supplementary file 3 — Figure S3. Monthly scan cluster analysis. Cluster 1 (circle with dash line in green color) and cluster 2 (circle with dash line in red color) were calculated with data for which denominators were imputed; cluster 3 (circle with dash line in purple color) and cluster 4 (circle with dash line in pink color) were calculated with no imputed denominators. Relative risks (RR) reflect the magnitude of the difference of the deaths in the cluster to regular deaths, which is the expected death assuming they occurred as driven by a homogenous Poisson process. This figure was made using a public domain map from the US National Park Service (https://www.nps.gov/hfc/carto/data-sources.cfm) as background. (PDF 97 kb) [file 12917_2018_1588_MOESM3_ESM.pdf]
