# Supplementary material for: Assessing changing weather and the El Niño Southern Oscillation impacts on cattle rabies outbreaks and mortality in Costa Rica (1985–2016)
Source: BMC Vet Res. 2018 Sep 17;14:285. doi: 10.1186/s12917-018-1588-8 (PMC6142330; doi:10.1186/s12917-018-1588-8)

Supplementary Figure S4 Correlation functions. **(A)** Auto-correlation function (ACF) of the monthly cattle rabies outbreak number time series; it shows the data were not strongly auto-correlated. **(B)** Partial auto-correlation function (PACF) of the monthly cattle rabies outbreaks time series data shows that there is a periodicity of 3 months. **(C)** Cross-correlation function (CCF) of the monthly cattle rabies outbreak number time series data with the ENSO4 index data. This CCF shows no significant linear association over the studied period, when considering up to 22 months of lag. **(D)** ACF of the monthly outbreak mortality time series. This time series shows low autocorrelation. **(E)** PACF of the monthly outbreak mortality time series. This time series shows a periodicity of 2 months. **(F)** CCF of the monthly outbreak mortality time series data with the ENSO4 index data. This CCF shows no significant linear association over the studied period, when considering up to 22 months of lag. In all panels blue dashed lines indicate the 95% confidence limits for correlations that can be expected by random.

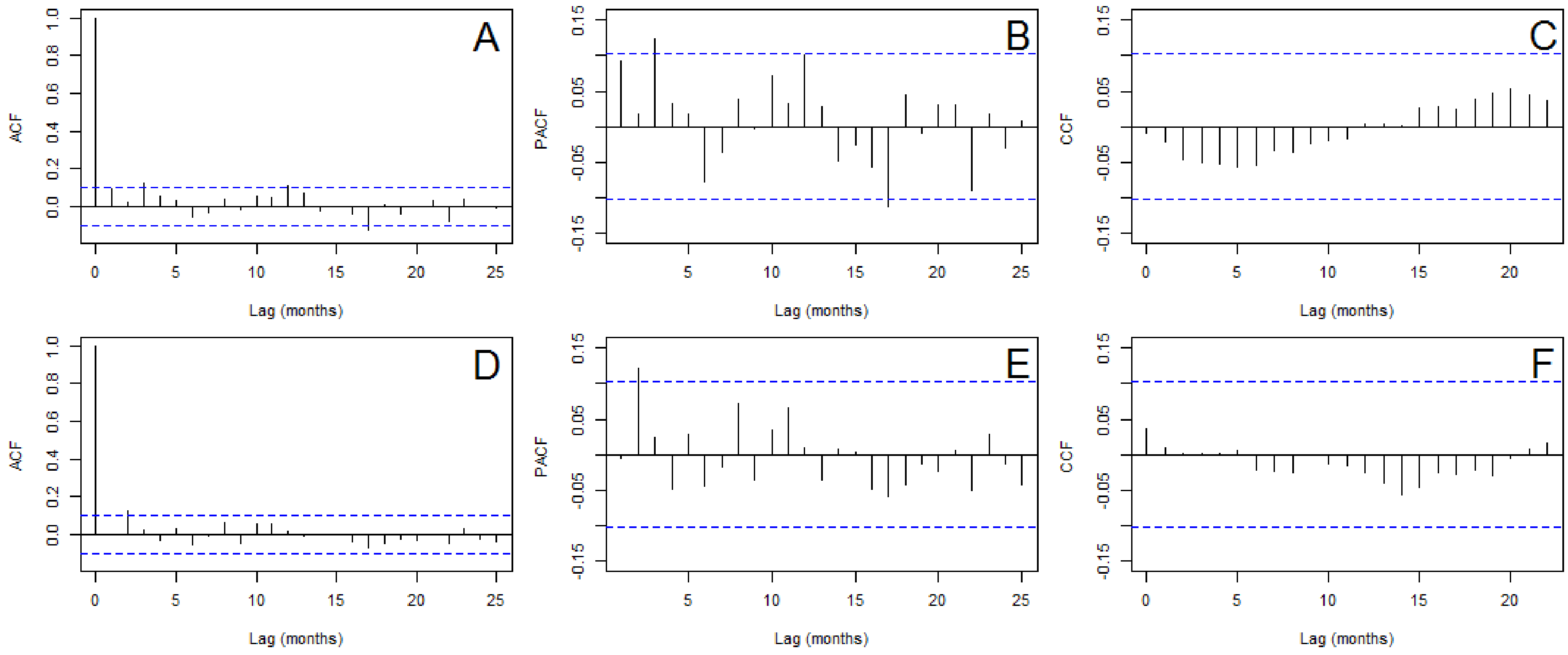

Supplement: Supplementary file 4 — Figure S4. Correlation functions. (A) Auto-correlation function (ACF) of the monthly cattle rabies outbreak number time series; it shows the data were not strongly auto-correlated. (B) Partial auto-correlation function (PACF) of the monthly cattle rabies outbreaks time series data shows that there is a periodicity of 3 months. (C) Cross-correlation function (CCF) of the monthly cattle rabies outbreak number time series data with the ENSO4 index data. This CCF shows no significant linear association over the studied period, when considering up to 22 months of lag. (D) ACF of the monthly outbreak mortality time series. This time series shows low autocorrelation. (E) PACF of the monthly outbreak mortality time series. This time series shows a periodicity of 2 months. (F) CCF of the monthly outbreak mortality time series data with the ENSO4 index data. This CCF shows no significant linear association over the studied period, when considering up to 22 months of lag. In all panels blue dashed lines indicate the 95% confidence limits for correlations that can be expected by random. (PDF 17 kb) [file 12917_2018_1588_MOESM4_ESM.pdf]
