# Supplementary material for: Assessing changing weather and the El Niño Southern Oscillation impacts on cattle rabies outbreaks and mortality in Costa Rica (1985–2016)
Source: BMC Vet Res. 2018 Sep 17;14:285. doi: 10.1186/s12917-018-1588-8 (PMC6142330; doi:10.1186/s12917-018-1588-8)
Supplement: Supplementary file 5 — Table S1. Selection of the best monthly cattle rabies outbreaks time series model. Columns indicate the type of model (models): full or the backward elimination round. The Akaike Information Criterion (AIC) is a model selection criterion which is minimized by the best model. The AIC for the best models of each selection round are bolded. o and x indicate, respectively, the presence or absence of a variable in a model. Temp is an abbreviation for temperature. Time lags are in months. (PDF 187 kb) [file 12917_2018_1588_MOESM5_ESM.pdf]

Supplementary Table S1 Selection of the best monthly cattle rabies outbreaks time series model. Columns indicate the type of model (models): full or the backward elimination round. The Akaike Information Criterion (AIC) is a model selection criterion which is minimized by the best model. The AIC for the best models of each selection round are **bolded**. o and x indicate, respectively, the presence or absence of a variable in a model. Temp is an abbreviation for temperature. Time lags are in months.

Models	Intercept	Covariates (Lag)						AIC
		Autoregressive (3)	Temp (17)	Temp (4)	Rain(16)	Rain (11)	Rain (3)	
Full	o	o	o	o	o	o	o	**590.29**
1st Round	o	o	o	o	o	o	x	589.36
	o	o	o	o	o	x	o	**588.30**
	o	o	o	o	x	o	o	589.81
	o	o	o	x	o	o	o	588.72
	o	o	x	o	o	o	o	596.06
2nd Round	o	o	o	o	o	x	x	587.41
	o	o	o	o	x	x	o	587.96
	o	o	o	x	o	x	o	**586.72**
	o	o	x	o	o	x	o	595.26
3nd Round	o	o	o	x	o	x	x	585.66
	o	o	o	x	x	x	o	587.44
	o	o	x	x	o	x	o	597.98
	o	o	o	x	x	x	x	**592.77**
	o	o	x	x	o	x	x	596.21
