# Supplementary material for: Assessing changing weather and the El Niño Southern Oscillation impacts on cattle rabies outbreaks and mortality in Costa Rica (1985–2016)
Source: BMC Vet Res. 2018 Sep 17;14:285. doi: 10.1186/s12917-018-1588-8 (PMC6142330; doi:10.1186/s12917-018-1588-8)
Supplement: Supplementary file 6 — Table S2. Selection of the best monthly cattle mortality time series model. Columns indicate the type of model: Null, or full model. The Akaike Information Criterion (AIC) is a model selection criterion which is minimized by the best model. The best model has its AIC bolded. o and x indicate, respectively, the presence or absence of a variable in a model. Temp is an abbreviation for temperature. Time lags are in months. (PDF 174 kb) [file 12917_2018_1588_MOESM6_ESM.pdf]

Supplementary Table S2 Selection of the best monthly cattle mortality time series model. Columns indicate the type of model: Null, or full model. The Akaike Information Criterion (AIC) is a model selection criterion which is minimized by the best model. The best model has its AIC **bolded**. o and x indicate, respectively, the presence or absence of a variable in a model. Temp is an abbreviation for temperature. Time lags are in months.

Models	Intercept	Covariates (Lag)		AIC
		Autoregressive (2)	Temp (17)	
Null Models	o	o	x	2522
Full	o	o	o	**2519**
